# Effect of Advanced Footwear Technology Spikes on Sprint Acceleration: A Multiple N-of-1 Trial

**DOI:** 10.1186/s40798-024-00758-w

**Published:** 2024-08-30

**Authors:** Benjamin Bernuz, Steven Laujac, Cedric Sirial, Stephane Auffret, Cristian Preda, Jean Slawinski, Benjamin Millot, Didier Pradon, Laure Coudrat, Olivier Gavarry

**Affiliations:** 1 Physical Medicine and Rehabilitation Departement, Neuro-Locomotor Day Hospital Unit, Leon Berard Hospital, Hyères, France; 2 DRCI, Centre Hospitalier Intercommunal Toulon-La Seyne sur Mer, Hôpital Sainte Musse, Toulon, France; 3 Les Fleurs Physiotherapy Center and Performance Division, Ollioules, France; 4 Athletic Beaussetan Club, French Athletics Federation (FFA), Comité du Var d’athlétisme, Toulon, France; 5 Biostatistics Department, Delegation for Clinical Research and Innovation, Lille Catholic Hospitals, GHICL, Lomme, France; 6 Laboratory Sport-Expertise and Performance (EA 7370), Research Department, French Institute of Sport (INSEP), Paris, France; 7 French Athletics Federation (FFA), Paris, France; 8 Pole Parasport - ISPC Synergies, UMR 1179 End:icap, INSERM Université Versailles-St-Quentin, CHU Raymond Poincaré, APHP, Garches, France; 9 Laboratory « Impact of Physical Activity on Health » (201723207F), University of Toulon, La Garde, France

**Keywords:** Advanced footwear technology, Super spikes, Sprint, Performance, Force-velocity profile, Overspeed, Muscle strain injury, N-of-1 trial, SCEDs

## Abstract

**Background:**

In contrast with Advanced Footwear Technology-AFT running shoes for long-distance, little is known about AFT sprint spikes on performance and acceleration parameters. However, their use has become widespread since the Tokyo 2020 Olympics, and knowledge of their effects would seem to be an essential starting point before any clinical or socio-economic considerations.

**Objectives:**

Our objectives were to determine intra- and inter-subject sprinting performance modifications with Nike^®^ AFT spikes (NAS) compared to standard spiked-shoes (SS).

**Methods:**

Healthy regional to national sprint athletes (*n* = 21, ≥ 750 pts World Athletics) performed 16 repetitions of 30-m sprints with either the NAS or SS condition during a single session, based on the multiple N-of-1 method, with pairwise randomisation and double-blind procedure. Time on 30-m sprints (Stalker radar), force-velocity profile (*F*_0_, *V*_0_, *V*_max_, *P*_max_, *RF*, *D*_RF_ and *FVP slope*), and confounding factors (wind and shoe mass) were measured. Statistical analyses included a mixed linear regression model for group analyses, and randomisation test inversion and non-overlap-of-all-pair (NAP) methods for intra-individual analysis.

**Results:**

NAS improved 30-m time by a mean of − 0.02 s (SMD = 0.4, *p* = 0.014), with no interaction with any confounding factors. Significant changes were seen in velocity (*V*_max_ : SMD = 0.9, *p* < 0.001; *V*_0_: SMD = 0.7, *p* < 0.001) and the horizontal ratio of force (*RF*_max_: SMD = 0.5, *p* = 0.043), with no changes observed in force production. Whatever the footwear, one unit of positive wind (+ 1 m.s^− 1^ ) improved performance by − 0.03 s (*p* < 0.001). At an individual level, four athletes improved (NAP ≥ 0.69), and one had a statistical decrease in performance. Changes in *F-V* profiles were largely individual.

**Conclusions:**

A positive effect on sprint acceleration characteristics was observed when using Nike^®^ AFT spikes, due to an increase in velocity and the horizontal ratio of force. A major variability in inter-individual response justifies single-case experimental designs for research on the topic.

**Trial Registration Number:**

NCT05881148.

**Supplementary Information:**

The online version contains supplementary material available at 10.1186/s40798-024-00758-w.

## Key Points

•Compared to AFT long-distance running shoes, little is known about trademarked AFT spikes on performance and acceleration parameters.

•The use of Nike^®^ AFT spikes in regional-to-national sprinters resulted in a slight improvement (less than that of a + 1 m.s^− 1^ wind) in short sprint performance, with a large effect on maximal velocity which could raise the question of muscle strain injuries.

•Trials addressing the question of AFT spikes’ effect on max velocity endurance should be designed. The significant rate of non-responders highlights the importance of single-case experimental studies.

**Supplementary Information:**

The online version contains supplementary material available at 10.1186/s40798-024-00758-w.

## Background

The advent of road racing shoes with advanced footwear technology (AFT) in 2016 was met with controversy and ethical concerns [1–4]. However, the research found a positive performance benefit when using AFTs on running economy and training performance for mid- and long-distance [5–8], so much so that several records have been set from the 5-km to the marathon [9]. AFT shoes combine “lightweight, resilient midsole foams with rigid moderators and pronounced rocker profiles in the sole” [10] and AFT spikes (also named “Super Spikes” [11]) made their debut at the Tokyo 2020 Olympics in the track disciplines, with these spikes now available to the general population. However, their effects on biomechanics and sprint performance are unknown and their price is high. Two recent retrospective studies suggested a positive effect, while a case-report highlighted some interesting underlying mechanisms [12–14]. However, high-quality direct evidence of improvements in biomechanics and associated performance is lacking. The 100 m race is arguably the highlight of the Olympic Games and World Championships, and as such has been the focus of much research [15]. It can be divided into three phases: acceleration phase, maximal velocity phase (*V*_*max*_), and the deceleration phase [16, 17], with research indicating that *V*_*max*_ depends primarily on the acceleration phase. In practical settings a force-velocity profile is used to understand the biomechanics of sprints, and has been validated against gold-standard methods [18–20]. Furthermore, the need for individualised answers is a growing concern. Standard parallel-group-designed trials have many limitations, as in the case of heterogeneous or small samples, and are not designed to give individualised results. Therefore, single-case experimental designs (SCEDs) have been described for some time [21, 22] and are now part of level 1 evidence-based medicine [23, 24]. They consist of multicycle crossover within-patient comparisons and allow for both individualised conclusions and generalisation of population issues.

The aim of our study was to compare the 30-m sprint performance between Nike^®^ AFT spikes (NAS) and standard spiked-shoes (SS) in inter-regional to national athletes, using the SCEDs method.

## Methods

### Participants

Athletes affiliated with the Var Athletics Committee (VAC) were screened using the SI-FFA software (Information System-French Athletics Federation). Inclusion criteria were as follows: healthy; ≥ 15 years-old; ≥ 750 World Athletics point level in sprinting (60–400 m); potential holder of a Nike Zoom Maxfly or Victory AFT spikes model for the NAS condition (own or borrowed). The Maxfly and Victory models are very similar, using the same compounds and the same layout in the midsole and outsole (see “Shoes information files”, supplemental material online, Fig. S1). The only differences are the mesh of the upper and the length of the rear plate (plastic sole and carbon plate along the entire length of the Maxfly, about half the length along the forefoot for the Victory). For the SS condition, their own standard spikes (with a stiff non-carbon plate, as EVA or PEBA) could be any brand. Forty-six eligible athletes and their coaches were contacted by email for verification and a newsletter. Twenty-five responded positively and 21 were available for one of the proposed half-day sessions. Some of the participants in the study were directly involved in the conception and design of the study, as were some of the coaches.

### Setting

The VAC Medical Commission, composed of doctors, physiotherapists, sports scientists, and FFA coaches, was in charge of the project in the Toulon area and Draguignan, France.

### Design

An N-of-1 trial is the generic term used for randomised double-blind SCEDs. It means that many comparison sequences (= N) between placebo (or standard of care treatment-A phase) and intervention (B-phase) will be performed on a single patient, leading to multicycle within-patient randomised double-blind crossover comparisons, that allow for intra-individual statistical analysis. The duration of phases and the number of measurements per phase may vary.

This N-of-1 was designed as an alternating randomised block design (aRBD) 1:1 [25], leading to multiple within-subject crossover comparisons of “NAS/SS” pairs (or cycle) randomly assigned (e.g. A/B/A/B/B/A/A/B/B/A/B/A…). The pairwise block allocation sequence was generated using a software program. The aRBD was chosen to respect the coaches’ and athletes’ preferences and constraints, allowing most sequence (= cycle) to be compared at the same time span. Due to the strict “on-off” intervention effect, no potential interference was expected between phases. We adhered to the N-of-1-specific “CENT” standards of reporting [26].

### Procedure

#### General Setting

**Fig. 1:**
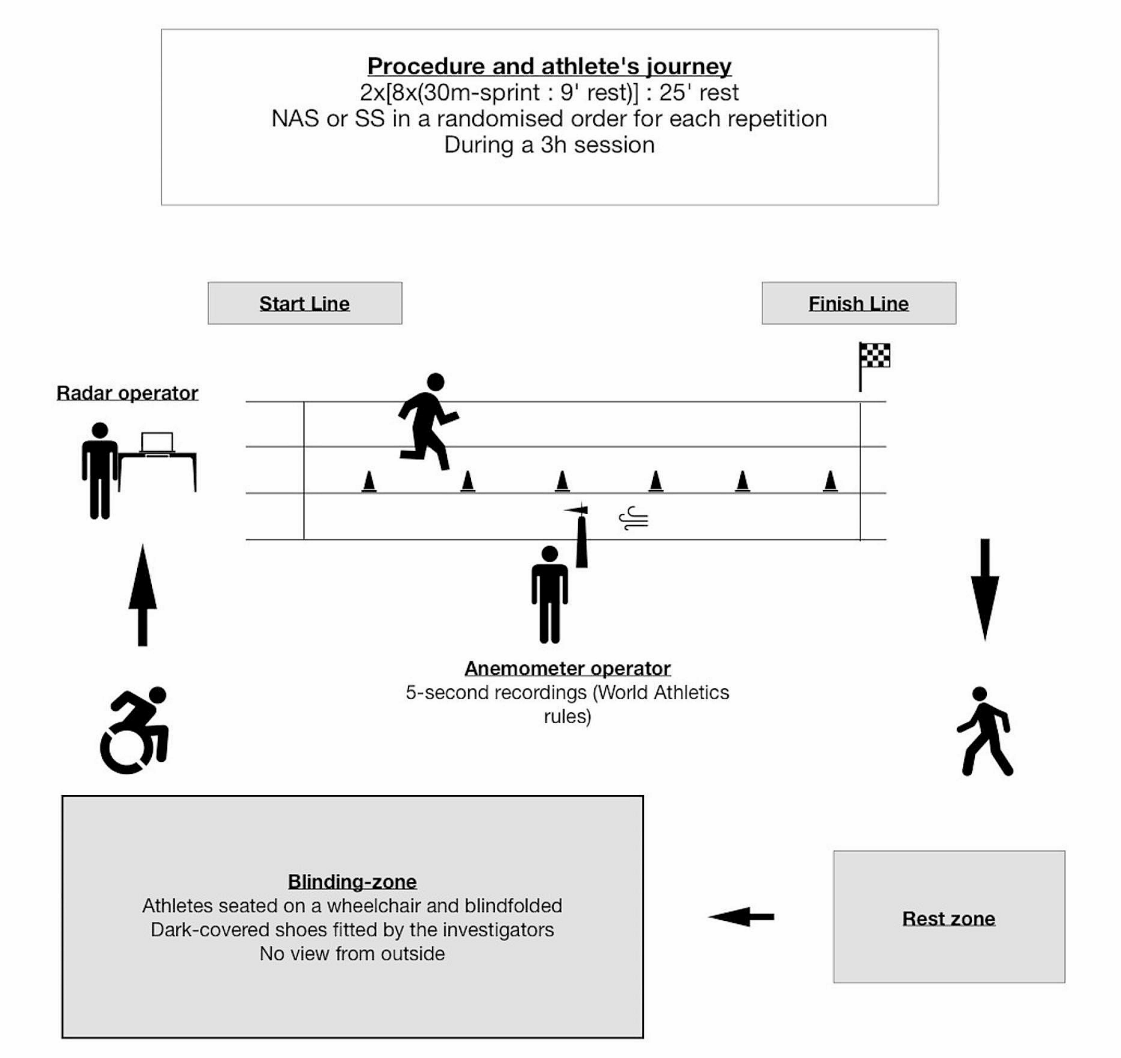
Global Procedure, pathway and athletes’ journey. *Note* Nike^®^ AFT Spikes (NAS), Standard spiked-Shoes (SS)

Each participant completed 16 repetitions (2 sets of 8) of 30 m sprints, performed at maximal effort, on the same track, either with NAS or SS, in a randomised order, during a 3 h total session (Fig. 1). The rest time between sets and repetitions was standardised (25 ± 5 and 9 ± 1 min respectively). The testing was preceded by a standardised 30 min warm-up comprised of 10 min low-intensity running, specific athletics drills, several submaximal accelerations with increasing intensity, and activo-dynamic stretching. A cooldown phase took place at the end. A split-stance starting position was adopted to limit the difficulties expected with starting blocks (i.e. setting changes and pelvic height for radar measurement). During the rest periods, water hydration was not controlled, with food and glucose allowed between two sets only (controlled). The wind was controlled for each repetition. Shoe weight was assessed and standardised when needed with the addition of one or several 20 g of ballast glued to the heel to decrease the weight difference. If the weight difference between shoe pairs was less than 40 g no weight was added to the shoe. We were unable to compensate for a weight difference of more than 100 g due to the lack of space to attach the ballast without inconveniencing the athletes. Altogether, only 6 athletes benefited from a shoe weight adjustment, with an average addition of 53 g (± 28) on shoe pairs. Among them, 2 adjustments were made on AFT (1 Maxfly, 1 Victory), while the other 4 were made on SS (see « Footwear conditions among participants », supplemental material, Table S1).

The 30-m distance was chosen to find the best compromise between evidence [16, 27] and feasibility (number of repetitions required and total distance volume), with a sufficient study time of the acceleration phase without the risk of the slowest athletes moving on to the next phase.

Data were collected and examined by two assessors who were blinded to the randomisation list.

### Blinding Method

A path was created between a blinding zone, and the start and finish lines. In this blinding zone, athletes were seated in wheelchairs, blindfolded, and fitted with shoes by the investigators. Both NAS and SS spikes were covered with dark socks during the warm-up and stored in numbered boxes inside the blinding zone. The steel spikes protruded from the socks. The socks were left on the spikes until the end of the session (athletes’ visual blinding; see supplemental material, Fig. S2). The investigators put the participant’s shoes on, to avoid any tactile information (“somesthetic blinding”). Shoes could not be seen by the assessors (assessors’ visual blinding) or by the other athletes in the blinding zone. Athletes were then taken to the start line with the wheelchair, to limit the difference in walking sensation caused by the NAS foam + air-pods (“proprioceptive blinding”). At the start-line for each repetition they were asked what shoe they thought they were wearing, NAS or SS, so that a ‘doubt’ rate could be assessed to check for the blind paradigm. This was defined as the percentage of the number of errors in response to the question: “What shoe do you think you are wearing, the AFT or the standard spikes?” in relation to the total number of trials. The blindfold was then removed, and the subject was allowed to stand and start. After the finish line there was a 20 m deceleration zone, and subsequently the sprinters sat back in the wheelchair, were blindfolded, and the shoes were removed and brought back to the blinding zone, as athletes entered the rest zone.

### Outcome Measures

#### Radar Measurement Process

During every maximal sprint, the instantaneous velocity was measured using a radar gun system (Stalker ATS II, Applied Concepts, USA), which is commonly known to provide reliable measures [28]. The radar was positioned 3 m behind the starting line on a tripod and set at the approximate height of each subject’s centre of mass.

The measured raw velocity-time data were cleaned with radar software (Stalker ATS 5.0) by deleting the data before the start and after 30 m, or identified as artefacts (data anomalies or inconsistencies identify such as peaks, losses, and noise by visual appreciation) to obtain the time at 30 m. Then, according to the validated model proposed by Samozino et al. [29], the horizontal velocity (*VH*) – time (t) curve was fitted using a mono-exponential function. This model allows for the calculation of the 30-m maximal velocity (*V*_*max*_) and the individual mechanical components of the force–velocity (*F*–*V*) relationship. The theoretical maximal force (*F*_*0*_) and velocity (*V*_*0*_) corresponding at the intersection of the *F*–*V* curve with the axis of force and velocity, as well as the slope of the linear *F-V* relationship (*F-V Profile* slope or *FVP slope*) were determined. Moreover, the individual ability to generate a greater amount of horizontal force was quantified as the force ratio (*RF*) of the net horizontal and vertical forces, therefore the maximal *RF* (*RF*_*max*_) and the decrease in *RF* (*D*_*RF*_) during the entire 30-m run were defined. The sprint data were processed using a spreadsheet (Microsoft Excel, USA) developed by Morin [30].

#### Confounding Factors

Wind was measured using the official World Athletics method, with 5-second recordings [31] (Anemometer SM-28, Speedtech Instruments, Virginia, USA, with a SpringCo tube and tripod support). Shoe masses were measured using a weight scale (PCB-6000, Kern ; Balingen, Germany).

### Statistical Analyses

All statistical analyses were performed using R statistical software (R Core Team, 2020). Continuous data were assessed for skewness by visual inspection of plots and normality tests. For all analyses, the probability was set at *p* < 0.05.

At the group level, an overall analysis of the 21 participants was performed to estimate the population effect using a mixed linear regression model (random intercept) and adjusting for covariates (confounding factors such as wind and shoe mass). An interaction was sought between the covariates that showed a statistically significant effect on the primary endpoint. Effect sizes (ES) were calculated using the standardised mean difference (SMD) method (Cohen’s d), as the mean difference estimated by the model relative to the pooled standard deviation (according to Cohen [32], SMD < 0.25 was considered trivial effect, 0.25–0.5 small effect, 0.5–0.8 moderate effect and > 0.8 large effect).

For intra-individual analysis in the framework of a randomised block single-case design (8 blocks, 2 conditions), for each participant, the unstandardised mean difference between the two conditions provided an estimate for the shoe-type effect. Confidence intervals and statistical significance were determined using a non-parametric randomisation test inversion methodology [33]. The non-overlap of all pairs (NAP) index [34] was used as the measure of the effect size, with values ranging as follows: small effect (0.5–0.66), medium effect (0.67–0.92), and large effect (≥ 0.93).

### Equity, Diversity and Inclusion Statement

The study population was chosen on the basis of sprint performance, regardless of their gender/ethnicity/socio-economic level. Marginalised groups were well represented, for example, with a majority of women and many people of colour. One of the aims of the study was to determine the added value of this footwear in relation to its additional cost, which seemed crucial for low-income populations. Socio-economic inequalities were erased by the possibility of wearing borrowed shoes for the test. The investigators’ team was multidisciplinary and gender balanced. The authors’ team included two junior researchers.

## Results

### Participant/Shoes’ Characteristics and Confounding Factors

A total of 327 of 336 30-m sprints (163 NAS/SS pairs) could be recorded, with only one missing data due to a failure to record, and four missing pairs because one patient dropped out after the first set (hamstring pain, 2 < VAS < 4 ; no hamstring injury was subsequently verified). Population and shoe characteristics are shown in Table 1.

Among the confounding factors, wind was the only factor that affects sprint time, in the entire group (−0.03 s, *p* < 0.001), without a significant interaction with shoe condition (wind x conditions, *p* = 0.36). Shoe mass (original or compensated), body-mass and height did not affect the sprint time.

**Table 1:** Population and shoes’ characteristics

**Population characteristics** Sex, mean (standard deviation) of the physical characteristics of the participants, athletic discipline (number of participants), median athletics scoring (min-max) and percentage (standard deviation) of errors in the subjective assessment of shoe-type during the session (= “doubt rate”, %).	
Sex (Female / Male)	14/7
Age (years)	19.2 (5.3)
Height (cm)	168.8 (6.7)
Body-mass (kg)	59.6 (8.8)
Athletic main discipline (n of participants)	S (16) H (2) LJ (1) TJ (1) PV (1)
Athletic level (World Athletics scoring)	863 (753-1001)
Errors in blind paradigm (%)	12.5 (14.5)
**Shoes characteristics** Mass (standard deviation) of the NAS versus SS; Number, ID distribution and size (median, min-max) of AFT-spikes among sub-types and participants.	
Original and compensated NAS pairs’ mass (g)	308 (74) / 313.5 (77)
Maxfly/Victory ratio	14/7
Victory users’ ID	3, 5, 8, 12, 17, 20, 21
Maxfly size (EU size)	40.5 (36-45)
Victory size (EU size)	40 (38-42.5)
Original and compensated SS pairs’ mass (g)	311 (54) / 320 (59)

Note H: hurdles; LJ: long jump; PV: pole vault; S: sprint; TJ: triple jump; EU: European; ID : athlete identification order; NAS : Nike AFT Spikes; SS : Standard spiked-Shoes

### At the Group Level

Sprint time was significantly lower in the NAS condition than in the SS condition, with a small ES (−0.02 s; SMD = 0.4, *p* = 0.014). This finding was associated with statistically significant: moderate-to-large ES on velocities (*V*_*0*_ and *V*_*max*_), moderate ES on the horizontal force ratio (*RF*_*max*_) and power (absolute and relative *P*_*max*_) under the NAS condition. However, the absolute and relative *F*_*0*_, *D*_RF_ and *FVP* values did not differ significantly between the two conditions (Table 2). There was no interaction between AFT subgroup conditions (Victory/Maxfly) and SS condition (SS x subgroups, *p* = 0.19).

**Table 2:** Estimated marginal mean differences in the main and secondary outcome measures during 30-m sprints

	SS	NAS	*p*	SMD
Sprint Time (s)	4.81 (0.05)	4.79 (0.05)	**0.014**	0.40
*V*_*max*_(m.s^-1^)	8.32 (0.09)	8.40 (0.09)	**< 0.001**	0.90
*V*_*0*_(m.s^-1^)	8.68 (0.11)	8.76 (0.11)	**< 0.001**	0.70
*F*_*0 absolute*_(N)	407 (7)	408 (7)	0.505	
*F*_*0 relative*_(N.kg^-1^)	6.84 (0.11)	6.85 (0.11)	0.656	
*RF*_*max*_(ratio)	48.3 (0.6)	48.6 (0.6)	**0.043**	0.50
*D*_RF_(ratio)	-7.20 (0.09)	-7.12 (0.09)	0.119	
*FVP*(ratio)	-0.790 (0.010)	-0.783 (0.010)	0.283	
*P*_*max absolute*_(W)	887 (23)	899 (23)	**0.006**	0.50
*P*_*max relative*_(W.kg^-1^)	14.9 (0.4)	15.1 (0.4)	**0.013**	0.50

*Note* bold p-values indicate significance for the difference between conditions; NAS : Nike AFT spikes; SS : Standard spiked-Shoes; the standardised mean difference (SMD) is added when the main effect of the condition is significant

### At an Individual Level

**Fig. 2:**
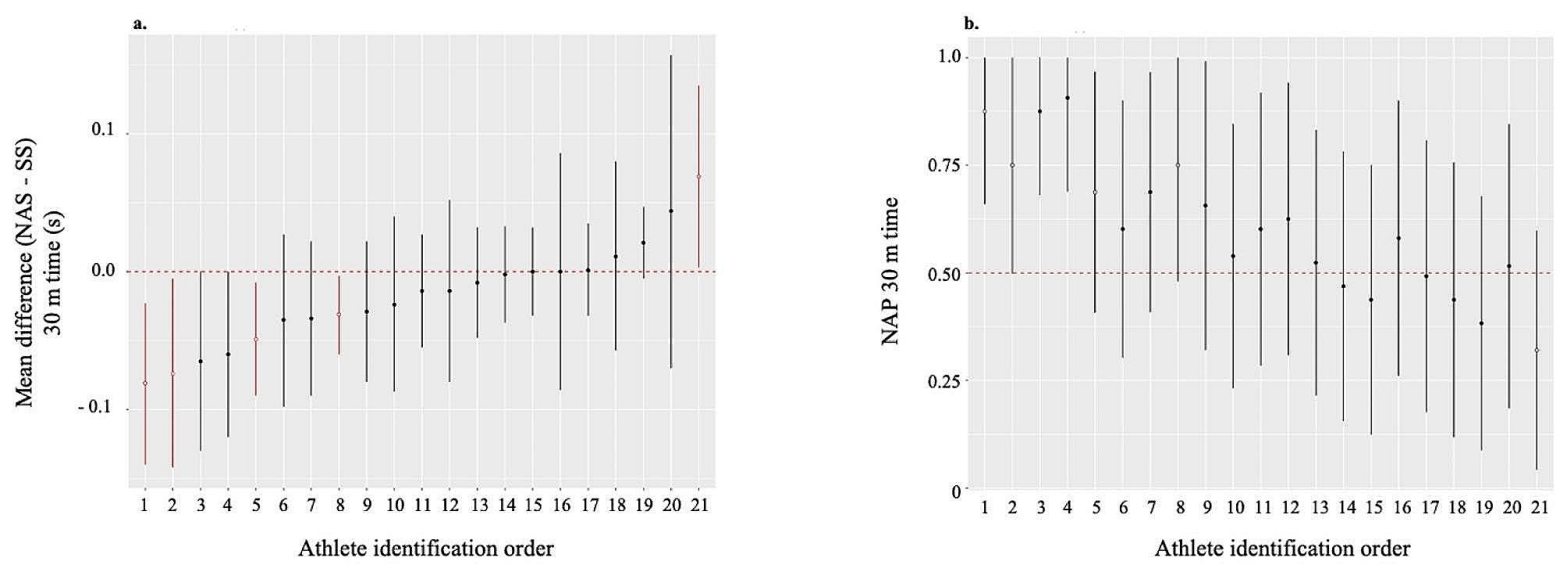
Mean difference and effect-size between NAS and SS for each athlete in 30-m time. **a**) Mean difference (± 95% CI) and **b**) effect size (± 95% CI) estimated by the non-overlap of all pairs (NAP) in 30-m sprint time between NAS and SS. *Note* Athlete identification in ascending order for 30-m time (s). Hollow circle and/or red colour for statistical significance. Nike^®^ AFT Spikes (NAS), Standard spiked-Shoes (SS)

**Fig. 3:**
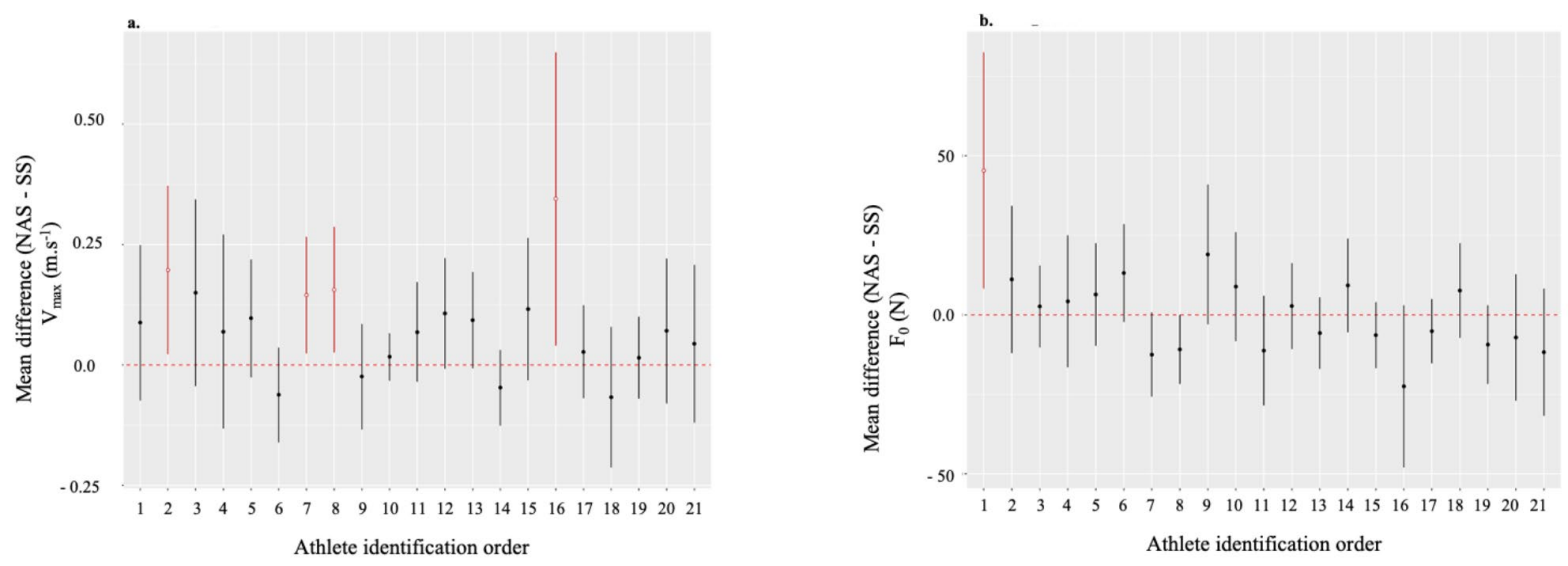
Mean difference in *V*_*max*_ and F_*0*_ between NAS and SS for each athlete. **a**) Mean difference (± 95% CI) in maximal velocity (*V*_*max*_) and **b**) mean difference (± 95% CI) in maximal horizontal force production (*F*_*0*_) between NAS and SS for each athlete. *Note* Athlete identification in ascending order for 30-m time (s). Hollow circle and/or red colour for statistical significance. Nike^®^ AFT Spikes (NAS), Standard spiked-Shoes (SS)

The subjects shown in the Figs. 2 and 3 are classified according to the estimated means of the main outcome criterion (30-m time), so that their identification number (ID) is always the same regardless of the figure.

#### The Main Outcome Measure

Only four subjects (2 females, 2 males) showed a statistically significant improvement in performance, leading to a mean difference ranging from − 0.03 to − 0.08 s (Fig. 2a). The corresponding effect-sizes were medium (NAP ranging from 0.69 to 0.87, Fig. 2b). Two were wearing the Maxfly model, and two were wearing the Victory model.

Conversely one subject’s performance (ID 21) was statistically worse (mean difference = + 0.07s; NAP = 0.32). He was wearing the Victory model and was the youngest.

The distribution of the 2 AFT models appeared to be balanced.

#### Secondary Outcome Measures

Five subjects showed at least one statistically significant change in one or more components of the *F-V profile* (sometimes with a large ES), including two subjects who did not benefit from a significant improvement in the main criterion. Several modifications of the *F-V profiles* occurred, but the vast majority were in favour of velocity increase (Fig. 3a). Only one subject (ID 1) showed an improvement in favour of *F*_*0*_ (Fig. 3b). No statistically significant modification of the *F-V Profile* components was observed to explain the deterioration in performance for subject ID 21.

## Discussion

To date, no experimental group study has compared sprint performance between AFT and standard spiked-shoes in athletes. Our main results clearly indicated that wearing a NAS improved 30-m sprint performance and acceleration parameters (medium-to-large effect on *RF*_*max*_ and *V*_*max*_). However, there was considerable variability in the inter-individual responses. The originality of our study lies in the methodological approach of using the SCEDs method to identify both individual and group changes in performance and biomechanical parameters of acceleration in athletes at the inter-regional to national levels.

### Clinical and Research Implications

#### Performance, Horizontal Force Application and Maximal Velocity Gains

The possible effect of wearing AFT spikes on sprint performance has recently been raised in the scientific literature. Two recent retrospective studies used an alternative approach, assessing changes in annual top 100 athletes’ performance, before and after the introduction of AFT spikes [12, 13]. Mason et al. [12] concentrated on sprint events and compared a 2016–2019 pre-AFT sprint spikes period with the 2021–2022 AFT era. The results suggest a 0.4 to 1.5% improvement during the AFT period, which was more pronounced for women and longer sprint distance. Willwacher et al. [13] compared a larger pre-AFT period (2010–2019) with the same AFT years. Improvements were estimated between 0.4 and 1.1%, again with better results for women and long distance races. In the present study, the significant gain in 30-m sprint performance was −0.02 s, or 0.4%, which is congruent with these findings and seems relevant in this context of a very short distance. If the gain in performance was to persist over 100 m, it would correspond to an improvement of −0.07 s, which is not negligible in a discipline where every hundredth of a second counts. Moreover, spiked shoes stiffened by isolated carbon plates (as an outsole or affixed to the inside of the outsole) have been available for some time, and many studies over the last 2 decades have focused on the effect of isolated longitudinal bending stiffness [34–37]. The vast majority of them failed to show a statistically significant positive effect on performance, despite some excellent results in some individuals. This underlines the importance of the combined technologies used in the NAS condition, with carbon plates combined with compliant/resilient foam (ZoomX) and air-pods (Air Zoom unit), all making up the midsole, whereas the SS condition consisted solely of an EVA/PEBA outsole without this AFT-specific midsole. In terms of the mechanisms underlying these modifications on performance, there is evidence suggesting the improvement is due to metabolic (running economy) factors in long-distance running [39], with an apparent advantage of PEBA over EVA before wear and tear [40], but there is very little data concerning sprint events. Our data clearly showed that the improvement in 30-m sprint performance using NAS was explained by the increase in *V*_*max*_ and *RF*_*max*_. The relationship between increased *RF*_*max*_ and improved sprint performance is known [41]. Our result of *RF*_*max*_ is consistent with a quasi-experimental case-report that found an increase in swing force and a decrease in vertical ground reaction force rate when assessing kinematics and kinetics between standard and AFT spikes in an Olympic-level sprinting athlete [14]. The decrease in vertical ground reaction force was preceded by a decrease in vertical impact, again suggesting the role of the foam/air-pod. The foam-mediated increase in midsole thickness, which is limited to 20 mm by World Athletics, may increase the lever arm and be another theoretical explanation for this forward swing. All these findings associated with the teeter-totter (rocking) effect of the stiff curved carbon plate [42] might illustrate the effect of this combination. In addition, it is important to note that the carbon plate is mounted on top of the midsole in the forefoot (see Supplementary files, Fig. S1), so that a ‘spring’ effect in the rearfoot at mid-stance could be added to the rocking effect (a spring-rocking effect).

*V*_*max*_ has also been found to be strongly correlated with sprint performance in previous literature [18, 43]. The 20 mm increase in midsole thickness may increase the overall limb length by 1–3% [12], as previous evidence suggests an association between longer length and faster velocity [44]. In our study, the group’s increase in *V*_*max*_ was 101%. Furthermore, some athletes showed statistically significant increases of 103 to 105% in *V*_*max*_, which is clinically relevant and share, for example, with the recommendations for overspeed-training intensity (103 to 110% of *V*_*max*_) [45]. Interest in studying what happens with these shoes beyond the first 30-m is highlighted by this positive effect on *V*_*max*_, which is an essential performance criterion beyond the acceleration phase [18, 43]. The combination of improved running economy seen during middle to long distance events, and the sprint specific biomechanical improvements seen in this study, may allow for improved performances in longer sprint events (200–400 m). Answering the question regarding the effect of these shoes on the ability to maintain *V*_*max*_ for as long as possible seems the next logical question.

#### Practical Implications: Individualised Answers, Cost-Benefit Ratio and Risk of Injury

First, some subjects were pole-vault, long-jump or triple-jump specialists. Thus, improvements in performance or speed at the end of the 30-m runway seem critical in simulating their real competitive run-up distance, and advice on shoe types was given to them.

Second, NAS shoes are more expensive than standard spiked-shoes (+ 275% annual average, recorded both on the Nike^®^ webstore and at a distance-selling site in September 2023 [46]). For a 100-m sprint, this could correspond to an additional cost of USD 20 per hundredth of a second obtained. In our study, the cost-effectiveness ratio could be calculated individually for each participant and helped some low-income subjects to make a choice.

Finally, the enhancement in speed is a key factor for performance, but it is also a factor in injury notably muscle strains. In particular, the high-speed running intensity has been highlighted [47]. Considering the high prevalence of hamstring strains injuries (HSI) in high-level athletics, an interesting follow-up to this study would be to examine this risk when using NAS. Moreover, it was recently shown that a decrease of 1 N.kg^− 1^ in horizontal force production (*F*_*0*_, initial acceleration) between two assessments in the season was associated with a 2.7 times greater risk of HSI in the following eight weeks in football players [48]. In our study, although *F*_*0*_ did not differ between the two conditions, some individuals showed a trend towards a clinically relevant decrease in their *F*_*0*_ production with the NAS in a single day (e.g. −0.18 to −0.36 N.kg^− 1^; *p* < 0.07). As recent studies have shown, there is a need for specific monitoring of this health problem, which depends on the biomechanics of sprinting and therefore on the use of AFT for sprinting [49].

### Methodological Considerations

First of all, it should be noted that at the time the study was designed in 2022, no brand other than Nike^®^ had marketed AFT spikes for sprinting in France.

Some factors should be considered when interpreting our findings.

The use of the 2 models of Nike^®^ AFT spikes in the intervention group could be a limitation. As explained in the “Methods” section, only the length of the rear plate (carbon and outsole plates) and the upper are different, although one model is lighter and more suitable for long-distance sprinters or middle-distance runners. With ‘the same concept, the same materials, the same layout, the same manufacturing process’, the decision was made to use both models of the Nike AFT spikes. However, this limitation did not change the interpretation of the intra-individual analysis, since each participant kept the same NAS model. In addition, there was no statistical difference between the two AFT subtypes in terms of time savings at the group level. For the “control” condition, the use of different footwear between participants could also be a methodological limitation. However, none of the shoes had the characteristics defined for an AFT [10]. In addition, as this was a single-case experimental-design study, it was necessary to be able to give an individual response based on the real life of each individual, with his/her own equipment.

It is important to also note the low rate of doubt introduced by the blind method (12.5%). In fact, 6 participants had a 0% doubt rate. These participants stated they could feel the difference when wearing the shoe. However, none of them were significantly improved by NAS. For the remaining 15 athletes, more uncertainty was observed.

The effects of these NAS compared with those of standard shoes were only studied over 30 m. To observe the true effect of the NAS it would be necessary to repeat the study over official distances such as 100 m. However, this N-of-1 method requires a minimum number of intervention/placebo cycles, which makes it difficult to assess a distance of 100 m due to fatigue. Designs other than alternating ones, such as ABAB which requires longer A/B phase with the same condition and fewer total repetitions [25] (i.e. not just one measurement per phase as in the alternating design, but several measurements during phase A, then several measurements during phase B on another day, then again at least one phase A and B), could be utilised to examine longer distances, but the downside is that it would not allow the sprints to be in the same time span.

Our study has several methodological strengths. First, the methodological approach used a randomised controlled SCEDs. In addition, confounding factors such as wind and shoe masses were considered in the statistical analysis. The use of the N-of-1 method proved very useful in providing factual answers to various individual questions, particularly because the effect for certain individuals was opposite to that of the group as a whole. Where applicable, this method clearly addresses some limitations of the group trials [50].

## Conclusion

This experimental study assessed for the first time the effects of AFT spikes on sprint performance and acceleration mechanical parameters in a group of state to national level sprinters. A small but significant gain in 30-m sprint performance is shown using advanced technologies marketed by Nike^®^, associated with a large effect on maximal velocity. The other components of the *F-V* profile were affected, but with considerable variability in the inter-individual responses. The use of the SCEDs methodology opens the way to individualised responses in terms of gains and cost-benefit / benefit-risk ratios.

### Electronic Supplementary Material

Below is the link to the electronic supplementary material.

Supplementary Material 1

Supplementary Material 2

## Data Availability

The datasets used and/or analysed during the current study are available from the corresponding author on reasonable request.

## Abbreviations

ABAB: Withdrawal/Reversal designs with sequences of alternating baseline (no-intervention or placebo-A) and intervention (B) phases
AFT: Advanced footwear technology
CENT: Consort extension for N-of-1 Trial
D_RF_: Decrease in Force Ratio of the net horizontal and vertical forces
ES: Effect size
EVA: Ethylene-vinyl acetate
FFA: Federation Française d’Athlétisme (French Athletics Federation)
F-V: Force-velocity relationship
F_0_: Theoretical maximal force of the Force-Velocity curve when V is 0
HSI: Hamstring strain Injury
NAP: Non-overlap of all pair
NAS: Nike advanced-footwear-technology spikes
PEBA: PolyEther block amide
RF_max_: Maximum force ratio of the net horizontal and vertical forces during the run
RBDa: Alternating Randomised Block Designs
SCEDs: Single-case experimental designs
SI-FFA: Système d’Information de la FFA (Information System-FFA)
SMD: Standardised mean difference
SS: Standard shoes
VAC: Var Athletics Committee (Comité d’Athlétisme du Var)
VAS: Visual analogue scale
V_H_: Horizontal Velocity
V_max_: Maximal Velocity
V_0_: Theoretical maximal velocity of the Force-Velocity curve when F is 0
