# Supplementary material for: Effect of Advanced Footwear Technology Spikes on Sprint Acceleration: A Multiple N-of-1 Trial
Source: Sports Med Open. 2024 Aug 30;10:92. doi: 10.1186/s40798-024-00758-w (PMC11364731; doi:10.1186/s40798-024-00758-w)
Supplement: Supplementary file 1 — Supplementary Material 1 [file 40798_2024_758_MOESM1_ESM.docx]

**Table S1. Footwear conditions among participants**

| **Participants ‘ ID** | **Sex** | **NAS sub-type** | **NAS size (EU size)** | **Used-NAS mass (g)** (mass added) | **Used-SS mass** **(g)** (mass added) |
| --- | --- | --- | --- | --- | --- |
| 1 * | M | Maxfly | 43 | 428 (+ 80 g) | 428 |
| *2 ** | M | Maxfly | 45 | 380 | 369 (+ 60 g) |
| *3* | F | Victory | 40 | 256 | 296 |
| 4 | F | Maxfly | 36 | 200 | 190 |
| 5 * | F | Victory | 40 | 256 | 286 |
| 6 # | F | Victory | 38 | 239 | 260 |
| 7 | F | Maxfly | 39.5 | 290 | 260 |
| 8 * | F | Victory | 40 | 250 | 355 |
| 9 # | M | Maxfly | 45 | 380 | 355 (+ 60 g) |
| 10 | F | Maxfly | 40.5 | 392 | 386 (+ 40 g) |
| 11 | M | Maxfly | 42.5 | 336 | 320 |
| 12 | F | Victory | 40 | 250 | 365 |
| 13 | F | Maxfly | 40 | 306 | 290 |
| 14 # | F | Maxfly | 42 | 530 (+ orth. Insole) | 420 (+ orth. Insole) |
| 15 | M | Maxfly | 41 | 326 | 351 |
| 16 | M | Maxfly | 43 | 350 | 366 |
| 17 | F | Victory | 38 | 240 | 292 |
| 18 # | F | Maxfly | 38.5 | 286 | 284 (+ 40 g) |
| 19 # | F | Maxfly | 38.5 | 285 | 274 |
| 20 | F | Maxfly | 38 | 282 | 258 |
| 21 * # | M | Victory | 42.5 | 319 (+ 40 g) | 320 |

*Note: EU: European; NAS : Nike AFT spikes; SS : Standard spiked-Shoes; F: female; M: male; Orth.:Orthopaedic; ID: participant identification number (classified according to the estimated means of the main outcome criterion (30-m time)) ;* : participant with a significant difference between NAS and SS ; # : participant with a 0% doubt-rate*
