# Supplementary material for: Effect of Advanced Footwear Technology Spikes on Sprint Acceleration: A Multiple N-of-1 Trial
Source: Sports Med Open. 2024 Aug 30;10:92. doi: 10.1186/s40798-024-00758-w (PMC11364731; doi:10.1186/s40798-024-00758-w)

**Fig. S1 Shoes additional information files : a) Maxfly model b) Victory model and c) Maxfly and Victory bottom view**

« The Maxfly and Victory models are very similar, using the same compounds and the same layout in the midsole and outsole » : AFT midsole with foam (ZoomX) and airpods (Air Zoom unit), embedded between a carbon plate (black plate) and the plastic outsole.

a) Maxfly model (full and forefoot side view):


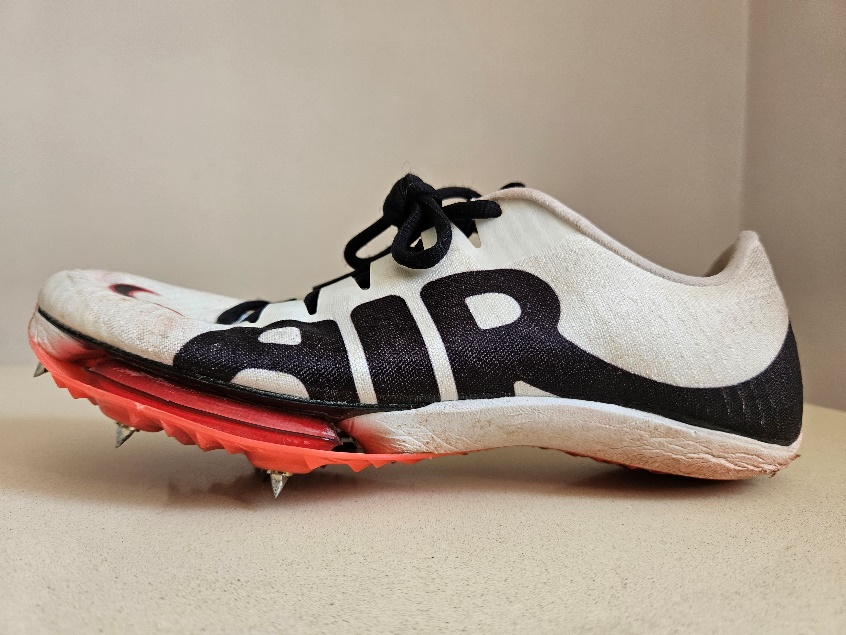

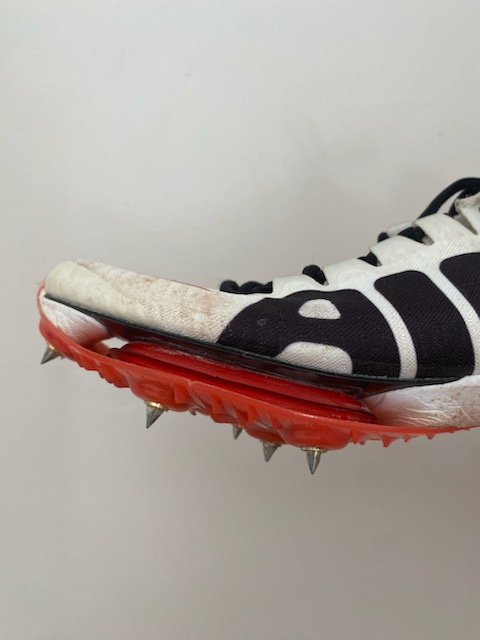


b) Victory model (full and forefoot side view)


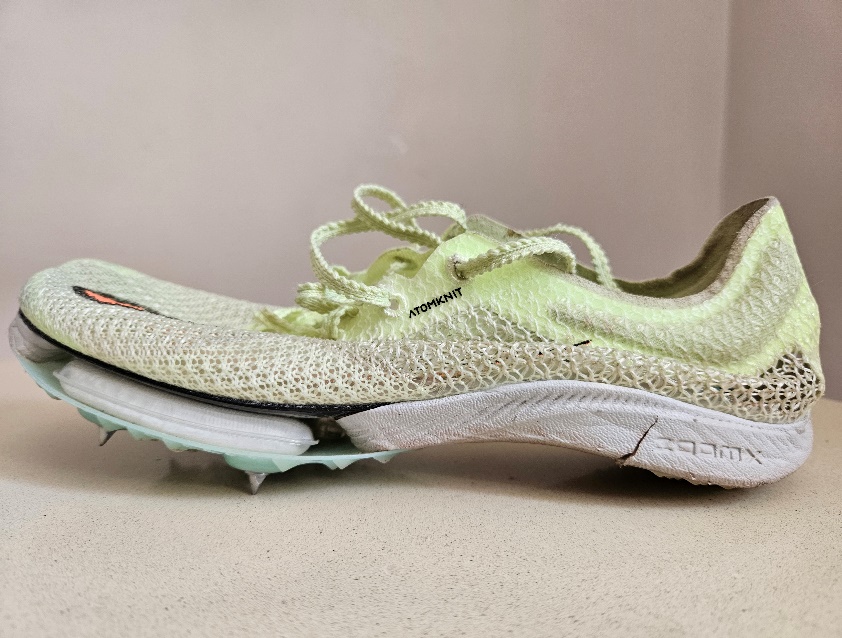

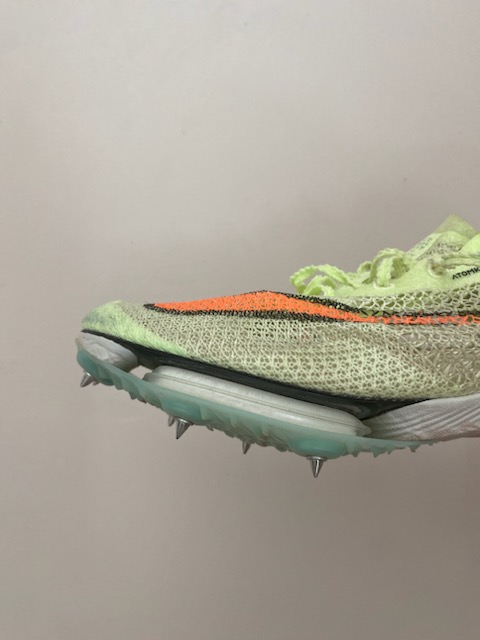


c) Maxfly and Victory (bottom view) :

“The only differences are the mesh of the upper and the length of the rear plate (plastic sole and carbon plate along the entire length of the Maxfly, about half the length along the forefoot for the Victory).”


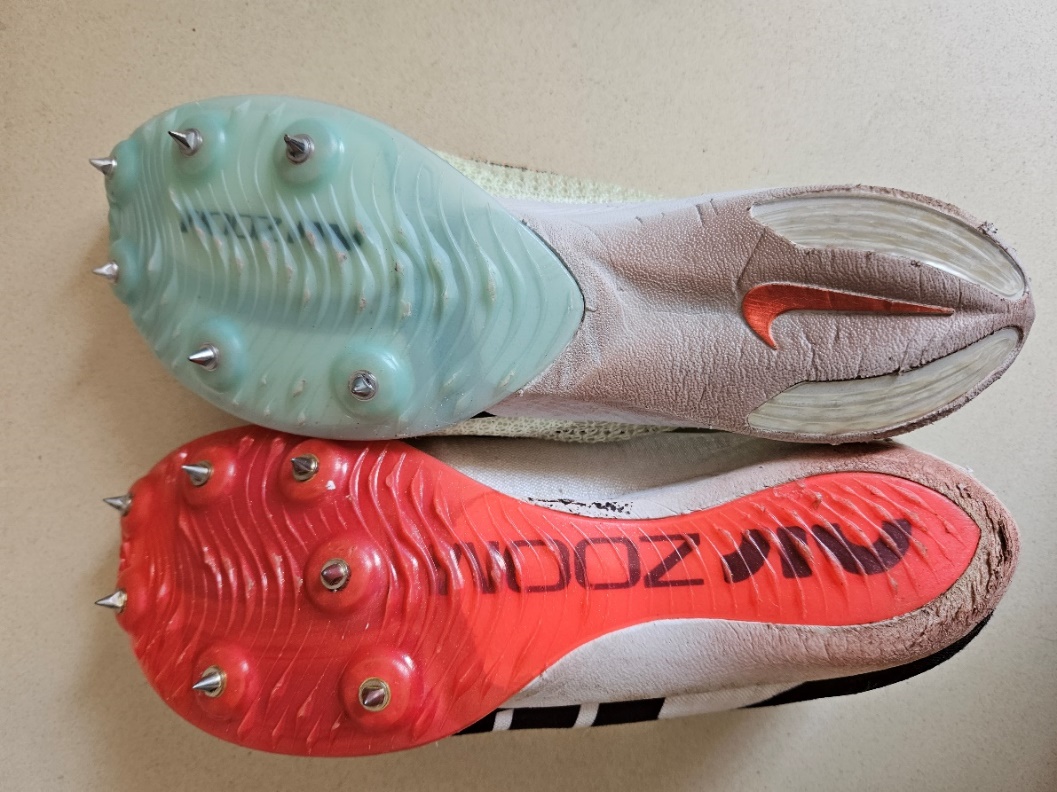


**Fig. S2 Blinding supplementary information: a) Exemple of a visual blinding b) « Spikes protruded » c) Example of a run**

a) Exemple of a visual blinding using NAS and SS models: « Athletes’ visual blinding : spiked-shoes covered with dark socks…»


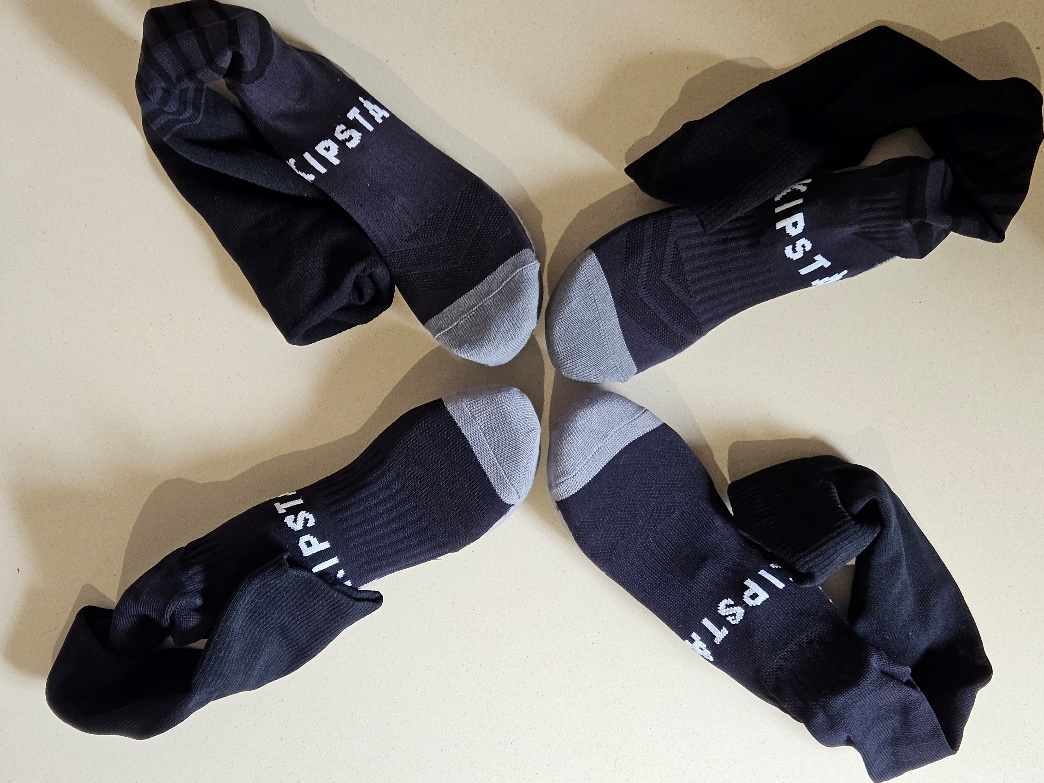


b) « The steel spikes protuded from the socks »


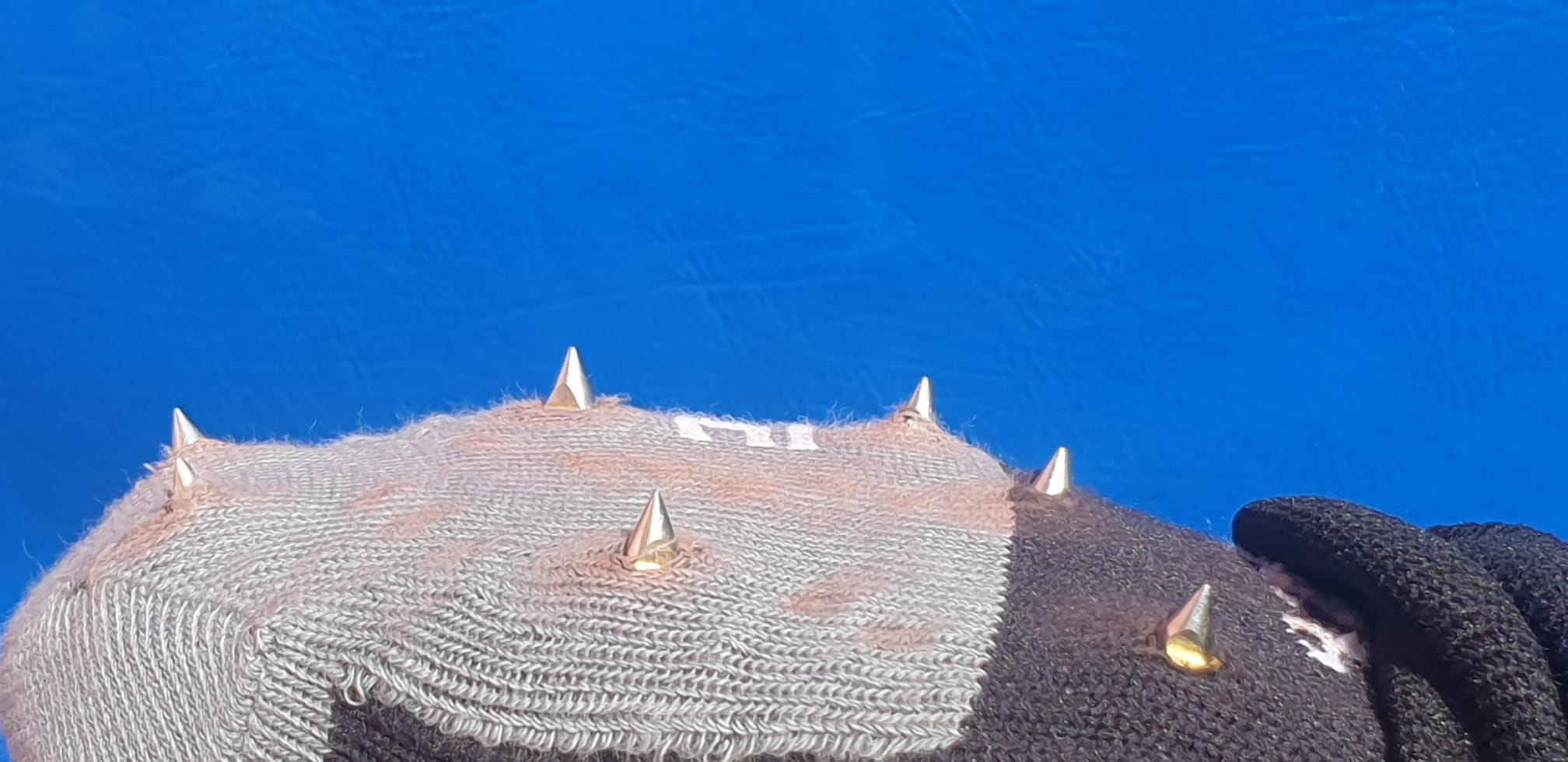


c) Example of a run


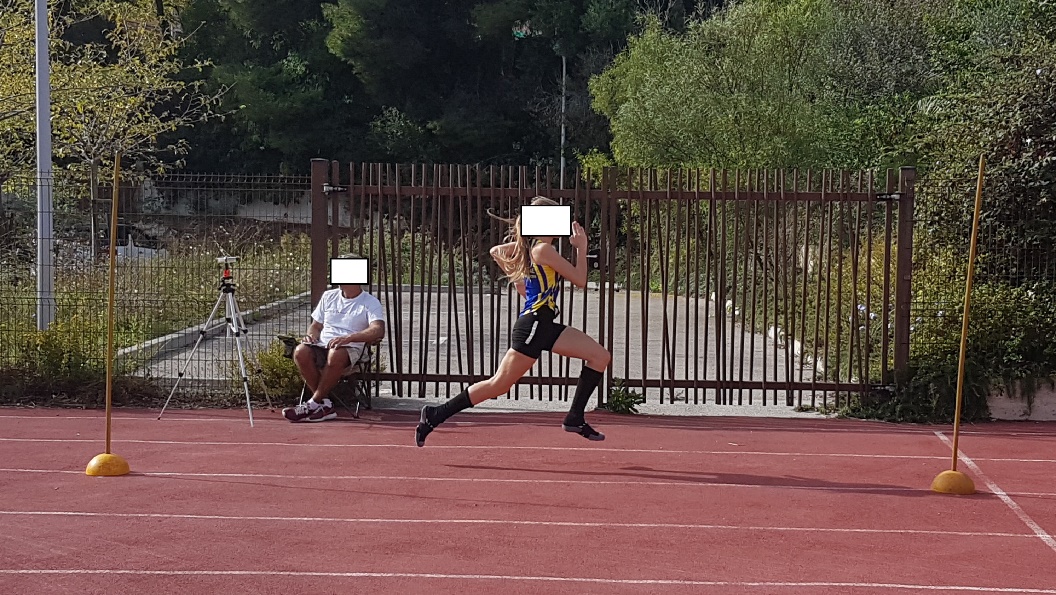

Supplement: Supplementary file 2 — Supplementary Material 2 [file 40798_2024_758_MOESM2_ESM.docx]
